# Prediction models for cardiovascular disease risk among people living with HIV: A systematic review and meta-analysis

**DOI:** 10.3389/fcvm.2023.1138234

**Published:** 2023-03-23

**Authors:** Junwen Yu, Xiaoning Liu, Zheng Zhu, Zhongfang Yang, Jiamin He, Lin Zhang, Hongzhou Lu

**Affiliations:** ^1^ School of Nursing, Fudan University, Shanghai, China; ^2^ Department of Infectious Diseases, National Clinical Research Center for Infectious Diseases, Shenzhen Third People's Hospital, Guangdong, China; ^3^ National Heart & Lung Institute, Faculty of Medicine, Imperial College London, London, United Kingdom; ^4^ Fudan University Centre for Evidence-Based Nursing: A Joanna Briggs Institute Centre of Excellence, Shanghai, China; ^5^ NYU Rory Meyers College of Nursing, New York University, New York City, NY, United States; ^6^ Shanghai Institute of Infectious Disease and Biosecurity, Fudan University, Shanghai, China; ^7^ Shanghai Public Health Clinical Center, Fudan University, Shanghai, China

**Keywords:** HIV, AIDS, cardiovascular disease, prediction model, systematic review, meta-analysis

## Abstract

**Background:**

HIV continues to be a major global health issue. The relative risk of cardiovascular disease (CVD) among people living with HIV (PLWH) was 2.16 compared to non-HIV-infections. The prediction of CVD is becoming an important issue in current HIV management. However, there is no consensus on optional CVD risk models for PLWH. Therefore, we aimed to systematically summarize and compare prediction models for CVD risk among PLWH.

**Methods:**

Longitudinal studies that developed or validated prediction models for CVD risk among PLWH were systematically searched. Five databases were searched up to January 2022. The quality of the included articles was evaluated by using the Prediction model Risk Of Bias ASsessment Tool (PROBAST). We applied meta-analysis to pool the logit-transformed C-statistics for discrimination performance.

**Results:**

Thirteen articles describing 17 models were included. All the included studies had a high risk of bias. In the meta-analysis, the pooled estimated C-statistic was 0.76 (95% CI: 0.72–0.81, *I*^2^ = 84.8%) for the Data collection on Adverse Effects of Anti-HIV Drugs Study risk equation (D:A:D) (2010), 0.75 (95% CI: 0.70–0.79, *I*^2^ = 82.4%) for the D:A:D (2010) 10-year risk version, 0.77 (95% CI: 0.74–0.80, *I*^2^ = 82.2%) for the full D:A:D (2016) model, 0.74 (95% CI: 0.68–0.79, *I*^2^ = 86.2%) for the reduced D:A:D (2016) model, 0.71 (95% CI: 0.61–0.79, *I*^2^ = 87.9%) for the Framingham Risk Score (FRS) for coronary heart disease (CHD) (1998), 0.74 (95% CI: 0.70–0.78, *I*^2^ = 87.8%) for the FRS CVD model (2008), 0.72 (95% CI: 0.67–0.76, *I*^2^ = 75.0%) for the pooled cohort equations of the American Heart Society/ American score (PCE), and 0.67 (95% CI: 0.56–0.77, *I*^2^ = 51.3%) for the Systematic COronary Risk Evaluation (SCORE). In the subgroup analysis, the discrimination of PCE was significantly better in the group aged ≤40 years than in the group aged 40–45 years (*P *= 0.024) and the group aged ≥45 years (*P *= 0.010). No models were developed or validated in Sub-Saharan Africa and the Asia region.

**Conclusions:**

The full D:A:D (2016) model performed the best in terms of discrimination, followed by the D:A:D (2010) and PCE. However, there were no significant differences between any of the model pairings. Specific CVD risk models for older PLWH and for PLWH in Sub-Saharan Africa and the Asia region should be established.

**Systematic Review Registration:** PROSPERO CRD42022322024.

## 1. Introduction

HIV continues to be a major global health issue. At the end of 2020, there were approximately 37.7 million people living with HIV (PLWH), with 1.5 million people becoming newly infected with HIV (1). The widespread usage of highly active antiretroviral therapy (HAART) drastically reduced death rates and potential years of life lost (2). However, as life expectancy increases, non-AIDS-defining illnesses are becoming increasingly common causes of death, with cardiovascular disease (CVD) accounting for a sizable portion (3, 4). CVDs, a set of heart and blood vessel disorders, are the leading causes of mortality worldwide. An estimated 17.9 million individuals died from CVDs in 2019, accounting for 32% of all global fatalities (5). According to a meta-analysis that included 793,635 participants from 73 studies, the relative risk (RR) of CVD among PLWH was 2.16 [95% confidence interval (CI), 1.68–2.77] compared to non-HIV-infections (6). In addition, it was indicated that HIV infection is a risk-enhancing factor for CVD (7). Moreover, ART may have a negative association with an increased risk of CVD events, especially among PLWH with other risk factors for cardiovascular disease (8, 9). As a result, the prediction and treatment of CVD are becoming even more important issues in current HIV management (4).

Prediction models are useful tools for estimating the probability or risk of specific future occurrences based on the combination of multiple predictors (10, 11). They aid clinicians in making therapeutic decisions and determining subsequent steps in therapy (12). CVD risk prediction is crucial to treatment guidelines and CVD control (13). Using prediction models is beneficial to make patients aware of their condition and to encourage them to adopt a healthy lifestyle (12). A substantial number of prediction models for various cardiovascular outcomes have been established for the general population, such as the Framingham Risk Score (FRS) (14), the Systematic COronary Risk Evaluation (SCORE) (15), and the pooled cohort equations of the American Heart Society/American score (PCE) (16). The usefulness of most of the models remains unclear because of methodological shortcomings, incomplete presentation, and lack of external validation (17).

Considering other potential factors driving CVD risk among PLWH, HIV-specific CVD prediction models have been developed, such as the Data collection on Adverse Effects of Anti-HIV Drugs Study risk equation (D:A:D) (18). Several CVD prediction models developed for the general population have also been validated in PLWH. However, the findings of model performance conflicted. Delabays et al. indicated that general models were valid to predict CVD for PLWH. Adding HIV-specific factors to scores did not result in a clinically significant improvement (19). In contrast, Triant et al. recommended adding HIV-related factors because general algorithms consistently underestimate the risk of CVD for PLWH (20). Whether HIV-specific models perform better than general models remains unknown. There is no consensus on optional CVD risk models for PLWH.

Soares et al. conducted a systematic review to summarize the CVD prediction models used for PLWH (21). However, they only considering studies published before January 31, 2021. Additionally, Soares et al. did not perform a subgroup analysis to ensure that the CVD risk prediction model treated subgroups (such as geographic region, race, and age) fairly in PLWH. The fairness of prediction models is crucial for promoting health equity, as ignoring differences in social determinants of health can result in inaccurate risk stratification in vulnerable groups, further exacerbating existing inequities (22).

Therefore, we aimed to systematically summarize multivariable prediction models for CVD risk among PLWH. Specific objectives included, first and foremost, objectively appraising the risk of bias in papers. Second, we conducted a meta-analysis on discrimination to estimate and compare the models' performance quantitatively. This review was conducted in accordance with the guidelines of the Checklist for critical Appraisal and data extraction for systematic Reviews of prediction Modelling Studies (CHARMS) (23). The protocol was registered on PROSPERO (CRD42022322024). The Preferred Reporting Items for Systematic Reviews and Meta-Analyses (PRISMA) guidelines were used to guide the reporting of our review (24).

## 2. Methods

### 2.1. Search strategy

We conducted a three-step search to identify both published studies and gray literatures. First, keywords and search terms were captured from an initial limited search *via* PubMed/MEDLINE. This informed the development of the search strategy with the help of a librarian. Second, a comprehensive search was conducted using the following databases: PubMed/MEDLINE, MEDLINE (Ovid), Embase (Ovid), CINAHL (EBSCO), and Web of Science. Gray literatures were searched *via* Google Scholar and Baidu. In PubMed/MEDLINE, we searched for papers in English using MeSH terms ([“HIV” OR “Acquired Immunodeficiency Syndrome” OR “HIV infections”] AND [prognosis OR survival OR mortality OR risk]) combined with the title/abstract keywords ([“progn*” OR “predic*” OR “risk” OR “model*”] AND [“HIV” OR “AIDS” OR “Acquired Immunodeficiency Syndrome” OR “PLWH”] AND [“machine learning” OR “artificial intelligence” OR “algorithm”] AND [“cardiovascular disease” OR “CVD” OR “cardiovascular event*” OR“main cardiovascular adverse event”OR “MACE”]). The search was limited to studies from inception to January 2022. The full search strategies for each database are presented in Supplementary Appendix S1. Finally, references in all included studies were manually reviewed to supplement the database search.

### 2.2. Inclusion and exclusion criteria

The inclusion criteria were as follows: (1) studies that assessed risk models that predicted the short-term or long-term risk of cardiovascular disease among PLWH. Cardiovascular disease in our review included coronary heart disease (CHD), myocardial infarction (MI), stroke, and other heart and blood vessel disorders (5). (2) Studies that developed new models, conducted external validation of an existing model, and/or updated an existing model. (3) Studies published in English or Chinese. No restrictions were made on the setting (e.g., inpatients, outpatients), prediction horizon (how far ahead the model predicted), or predictors or outcomes. Cross-sectional studies were excluded. Studies that merely carried out risk factor analysis without modeling were further excluded.

### 2.3. Study screening and selection

All identified citations were imported into EndNote X8 (Clarivate Analytics, PA, United States) to remove duplicates. Two reviewers (JY & ZZ) independently carried out screening and selection. First, titles and abstracts were screened to ascertain potentially relevant studies. Next, we screened the full texts and identified studies that met the inclusion and exclusion criteria. Discrepancies were discussed between two reviewers until a consensus was reached after referring to the protocol.

### 2.4. Quality appraisal

We appraised the quality of the included articles by using the Prediction model Risk Of Bias ASsessment Tool (PROBAST), which is an assessment tool developed specifically for diagnostic and prognostic prediction model studies (25). Two reviewers (JY & ZZ) assessed the risk of bias and applicability respectively. For the assessment of risk of bias, four domains (participants, predictors, outcome, analysis) with a total of twenty signaling questions were judged. Each signaling question was answered as “yes”, “probably yes”, “probably no”, “no”, or “no information”. Based on the answers to the signaling questions, we used our own judgment to rate the domains as high (−), low (+), or unclear (?) risk of bias. We assessed applicability by using the same first three domains but without signaling questions. Finally, we rated the overall ROB and applicability as high (−), low (+), or unclear (?). Any disagreement was discussed between the two reviewers until a consensus was reached.

### 2.5. Data extraction

Two reviewers (JY & ZZ) independently extracted information from the included papers, including the authors, year, location, study design, study population, predicted outcomes, predictors, sample size, missing data, modeling method, method of validation, and predictive performance. We extracted data separately for each model when a study described multiple models. Any disagreement was discussed, and a consensus was reached.

### 2.6. Data synthesis

We performed descriptive analysis to summarize the characteristics of the models. If a model was validated in two or more studies, we applied a random effects meta-analysis model to evaluate discrimination performance (26). Since different studies reported different discrimination metrics, either by Harrell's C-statistic or by area under the curve (AUC), we further pooled them separately. Due to the variability in the validation population, differences in outcome and predictor definitions across studies, and the non-normal distribution of the C-statistic between studies, we transformed the C-statistic using a logit transformation (27). When confidence intervals were not available, we approximated them by using the standard normal distribution (28). We considered C-statistics in the range of 0.50–0.59 to indicate poor, 0.60–0.69 to indicate moderate, 0.70–0.79 to indicate acceptable, 0.80–0.89 to indicate very good, and 0.90 or greater to indicate excellent discrimination (29). The *I*^2^ statistics were used to quantify the heterogeneity of the studies. In addition, subgroup analyses were performed in terms of sample size, age, and follow-up. The validation cohort was defined as “Mainly White” if more than 50% of the population was white. Since all the studies encompass both white and black individuals, the term “Mainly” was used instead of specifying either “White” or “Black”. Analyses were performed in Stata 17.0 (College Station, TX).

## 3. Results

### 3.1. Inclusion of studies

**Figure 1:**
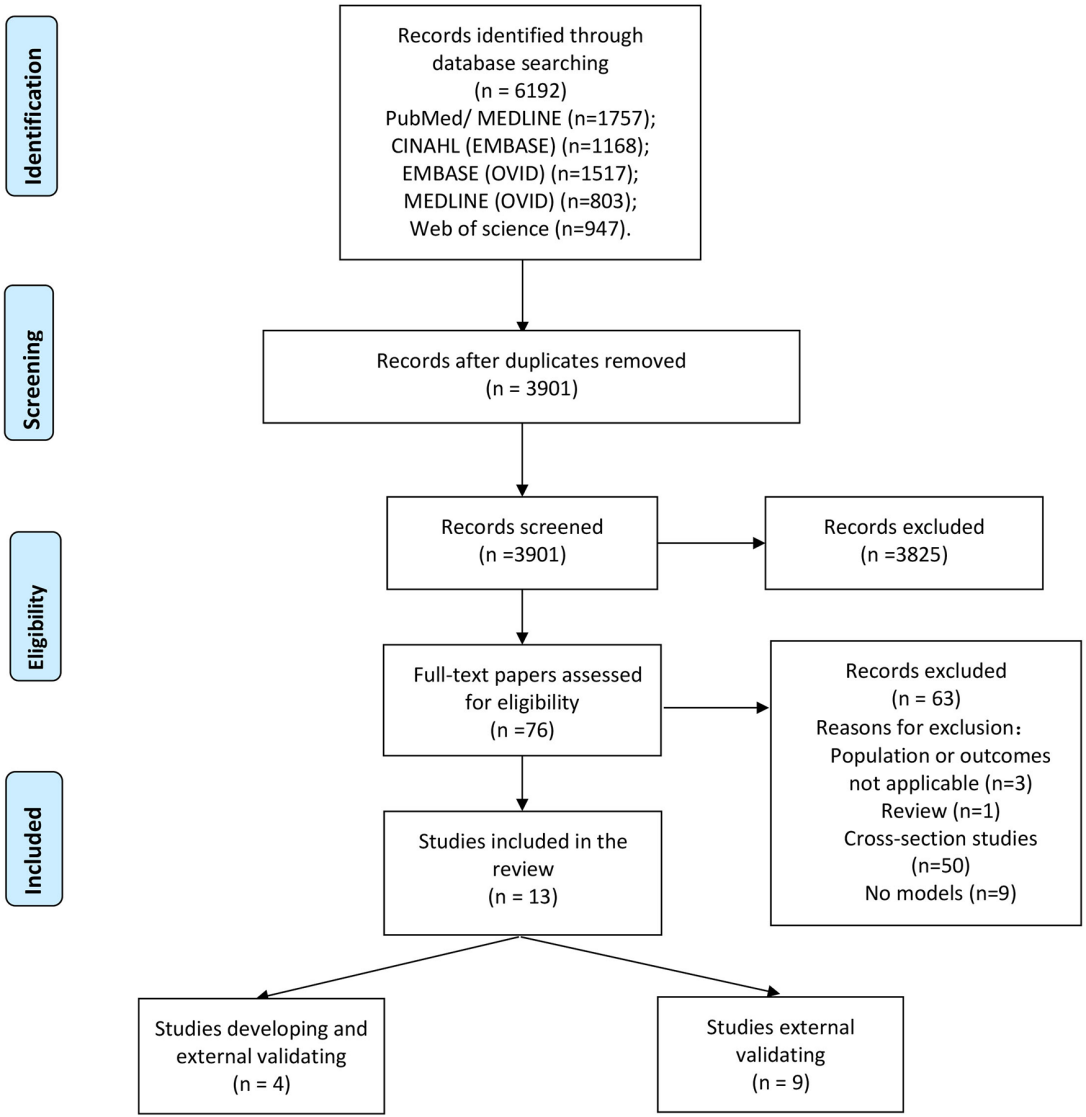
Flow diagram of the selection process.

A total of 6,192 records were obtained from the databases. After removal of duplicates, the titles and abstracts of 3,901 articles were screened. The full texts of 76 articles were reviewed, and 63 articles were excluded because they did not meet the predefined inclusion criteria. Finally, 13 articles were included in our systematic review (Figure 1) (19, 20, 30–40).

### 3.2. Characteristics of eligible studies

Table 1 shows the characteristics of the included studies. Overall, 17 different models were derived from 13 studies (19, 20, 30–40). There were six HIV-specific models, including the D:A:D model (2010), the D:A:D (2010) 10-year version model, the updated full D:A:D (2016) model, the updated reduced D:A:D (2016) model, the HIV MI-1, the HIV MI-2 models. The other 11 models were developed for the general population and validated in PLWH, including the FRS (2004), FRS for CVD risk (2008), FRS for CHD risk (1998), FRS adjusted for Colombia, FRS adaptation for the Spanish population (REGICOR), FRS developed specifically for the Italian population by Progetto CUORE, the Prospective Cardiovascular Munster Study (PROCAM), SCORE, SCORE 2, SCORE adjusted for national data (SCORE-NL), and PCE.

**Table 1:** Characteristics of the included studies.

Author, Year	Country	Study Design	Sample Size	Age (years)	Male	Follow-up	Number of Outcome Events
Anikpo, 2021	US	Prospective cohort Validation only: D:A:D (2016) reduced model	1,029	Median: 45.0 (35.0–52.0)	70%	Baseline: 2 years; Follow-up until 2019.12.31	78
Delabays, 2021	Switzerland	Prospective cohort Validation only: D:A:D (2010), SCORE2, PCE	6,373	Mean: 40.6 ± 9.0	71.60%	Baseline: 7 years; Mean follow-up time: 13.5 ± 4.1 years	533
De Socio, 2017	Italy	Prospective cohort Validation only: FRS CVD (2008), SCORE, Italian “Progetto Cuore”	369	Mean: 43.0 ± 9.0	62.90%	Baseline:: 1 year; Median: 10 years	34, 9, 21, respectively
Feinstein, 2017	US	Prospective cohort Development: HIVMI-1, HIVMI-2; Validation: PCE	HIVMI: 19,829, PCE: 11,288	Mean: 41.3	81.90%	Mean follow-up: 4.1 years, censored at 10 years	HIVMI: 353, PCE: 247
Friis-Møller, 2010	Europe, Australia	Prospective cohort Development: D:A:D (2010) Validation: FRS CHD (1998), FRS CVD (2008)	22,625	Median: 40.0 (35.0–47.0)	74.10%	Median baseline: 2000.7 Median follow-up years: 4.80 (3.04–7.00) years	CVD/CHD/MI events: 663/554/387
Friis-Møller, 2016	Europe, Australia	Prospective cohort Development: D:A:D (2016) full and reduced model Validation: FRS CVD (2008)	32,663	Median: 39.0 (33.0–46.0)	74%	Median follow-up time: 5.7 (2.9–8.8) years Follow-up until 2011.2.1	1,010
García-Peña, 2021	Colombia	Prospective cohort Validation only: D:A:D (2010), FRS (2004), FRS CVD (2008), FRS Adjusted for Colombia, SCORE, PROCAM, PCE	808	Mean: 35.0	88%	At least 5 years	8
Herrera, 2016	Spain	Prospective cohort Validation only: FRS CHD (1998), REGICOR	641	Mean: 45.7 ± 9.5	81.65%	Baseline: 0.5 year Follow-up until 2013.6 Mean follow-up: 10.2 years	38
Raggi, 2016	Italy	Prospective cohort Validation only: DAD (2016), FRS CVD (2008), PCE	2,550	Median: 45.0 (42.0–49.0)	66%	Mean follow-up: 6.5 years	69
Schulz, 2021	Germany	Prospective cohort Validation only: FRS CHD (1998), SCORE, PCE	626 totally; 470, 567, 464, respectively	Mean: 53.0, 53.0, 53.0, respectively	89.40%, 89.20%, 89.70%, respectively	Mean follow-up time: 4.5, 4.6, 4.6 years, respectively	57, 44, 46, respectively
Thompson-Paul, 2016	US	Prospective cohort Validation only: D:A:D (2010), D:A:D (2010) 10-year version, FRS CVD (2008), PCE, SCORE	2,283	Median: 42.2 (36.4–48.4)	75.90%	Baseline: 8 years; Censored: 2013.9.30 Median follow-up 6.6 (3.3–10.4) years	220, 199, 151, 18, respectively
Triant, 2018	US	Prospective cohort Validation: FRS CHD (1998), FRS CVD (2008), PCE; Developed new models based on HIV cohort	1,272	Mean: 51.2 ± 8.7	100%	Baseline: 3 years; Median follow-up time: 4.4 years	hard CVD: 48; ASCVD: 78
van Zoest, 2019	Netherlands	Prospective cohort Validation only: D:A:D (2016), D:A:D (2010) 10-year version, SCORE-NL, FRS CVD (2008), PCE	16,043, 15,986, 15,866, 16,070, respectively	Median: 43.0 (36.0–50.0)	82.40%	Median follow-up: 5.4 (2.5–9.0), 5.4 (2.5–9.0), 5.3 (2.5–8.9), 5.4 (2.5–9.0) years Censored at 10 years at the earliest	478, 1,138, 1,393, 955

D:A:D, Data collection on Adverse Effects of Anti-HIV Drugs Study risk equation; FRS, Framingham Risk Score; REGICOR, FRS adaptation for the Spanish population; PROCAM, the PROspective CArdiovascular Munster study; SCORE, Systematic COronary Risk Evaluation; SCORE-NL, SCORE adjusted for national data; PCE, pooled cohort equations of the American Heart Society/American score.

In total, four studies (20, 32–34) described the development process of prediction models, and 13 studies (19, 20, 30–40) conducted model external validation. Four studies were carried out in the United States (20, 30, 32, 39), followed by Italy (*n* = 2) (31, 37), Switzerland (*n* = 1) (19), Colombia (*n* = 1) (35), Spain (*n* = 1) (36), Germany (*n* = 1) (38), and Netherlands (*n* = 1) (40), with two being multicenter studies from several countries (33, 34). The sample size ranged from 369 (31) to 32,663 (34). The number of prediction events varied from 8 (35) to 1,393 (40). Twelve studies were mostly conducted on males, while one study (20) included only male participants.

Among four development studies, three (20, 33, 34) used backward selection to remove nonsignificant predictors, whereas the other one (32) did not report a specific strategy for variable selection. Strategies dealing with missing data varied, including excluding participants with missing data (34) and using single imputation (32); one study did not describe a strategy (33).

Among 13 validation studies, nine studies reported both the calibration and discrimination performance of the models (19, 20, 30–34, 39, 40). Two studies reported only discrimination (35, 38), one study reported only calibration (37), and one study did not report model performance (36). Discrimination was reported as Harrell's C-statistic in six studies (20, 30, 34, 39, 40) and AUC in five studies (19, 31, 33, 35, 38). A variety of methods were used to report the calibration performance of the models, including the observed-expected ratio (*n* = 5) (30, 31, 33, 39, 40), Hosmer–Lemeshow test (*n* = 3) (19, 34, 39), calibration plot (*n* = 3) (30, 32, 40), Greenwood-Nam-D'Agostino (GND) test (*n* = 3) (20, 32, 40), and Brier score (*n* = 1) (19).

### 3.3. Risk of bias

**Figure 2:**
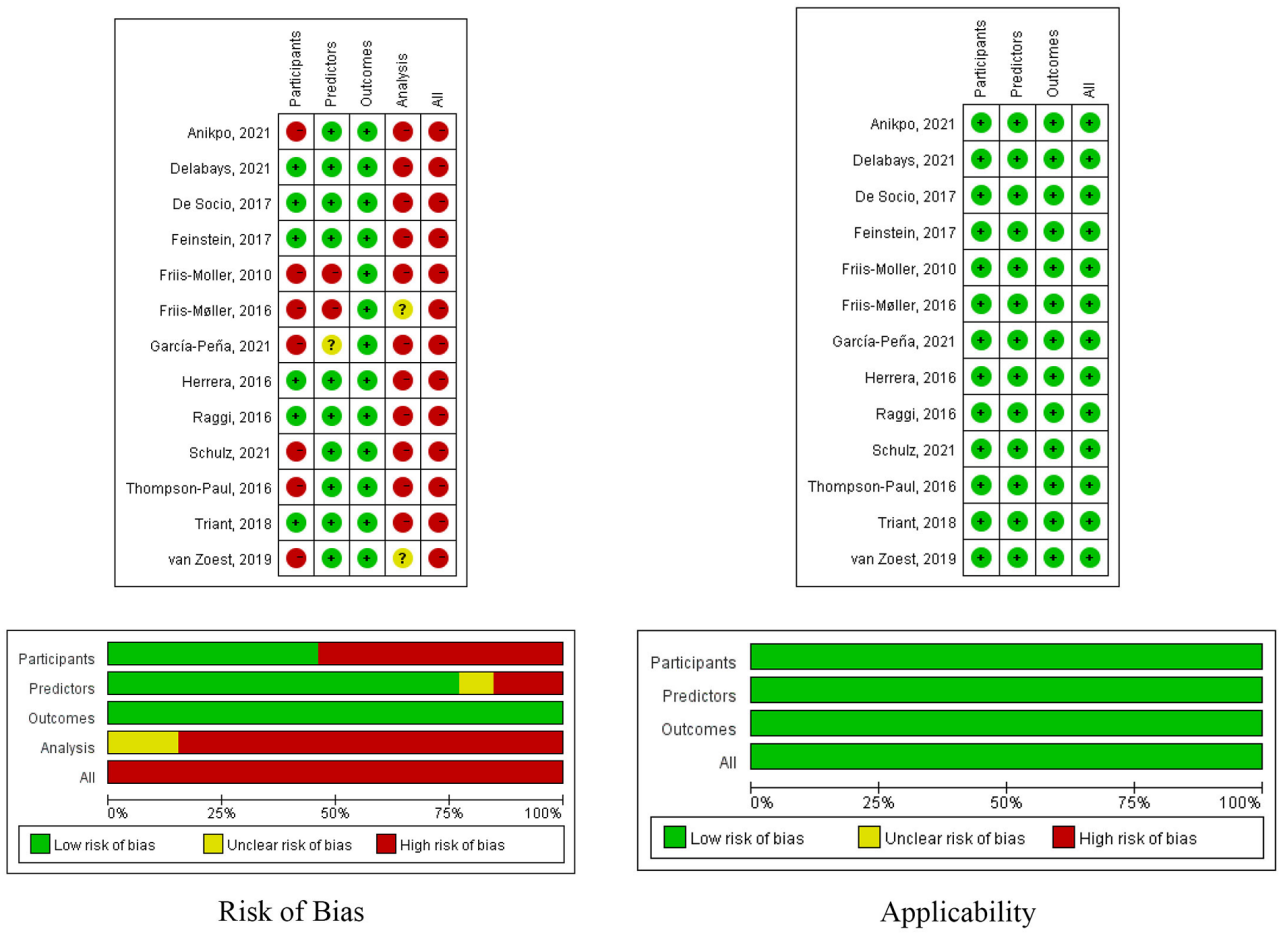
Risk of bias and applicability of the included studies.

Figure 2 shows a summary of the risk of bias and applicability for all included studies. All of the included studies had a high risk of bias. Ten studies (76.9%) (19, 20, 30–32, 36, 37, 40) were considered to have a low risk of bias in the predictor domain, and all the included studies (19, 20, 30–40) were considered to have a low risk of bias in the outcome domain. The risk of bias related to the participant domain was high in seven studies (53.8%) (30, 33–35, 38–40), mainly because they excluded participants with missing data. Twelve studies were judged to have high risk of bias in the analysis domain (19, 20, 30–33, 35–40). Nine studies ignored or dealt with model overfitting in an inappropriate manner (18, 30–32, 35–39). Seven studies had lower-than-reasonable numbers of participants with the events (20, 30, 31, 35–38). Ten studies did not report the methodologies for continuous and categorical predictor transformation (19, 20, 31, 32, 35–40).

### 3.4. HIV-specific models

Table 2 shows the characteristics of six HIV-specific models (32–34, 39, 40). In total, four models were developed in multicenter studies (33, 34, 39, 40), while the remaining two models were developed in the US (32). Four models could be used in all age groups (33, 34, 39, 40). Three models were developed to predict the 5-year risk of CVD, including the D:A:D (2010), updated full D:A:D (2016) and reduced models, whereas the other three models were for 10-year risk prediction. For the modeling methods, three models utilized Cox proportional hazards modeling (32, 34), two used Poisson regression modeling (33, 39, 40), and one used lasso and ridge regression (32).

**Table 2:** Predictors and outcomes of six HIV-specific models.

		D:A:D (2010) for 5-year risk (33)	D:A:D (2010) for 10-year risk (39, 40)	Full D:A:D model (2016) for 5-year risk (34)	Reduced D:A:D (2016) model for 5-year risk (34)	HIV MI-1 (32)	HIV MI-2 (32)
**Population**	Geographic region	Europe, Argentina, Australia, US	Europe, Argentina, Australia, US	Europe, Argentina, Australia, US	Europe, Argentina, Australia, US	US	US
	Age limits	Not defined	Not defined	Not defined	Not defined	40–79 years old	40–79 years old
**Predictors**	Age	√	√	√	√	△	△
	Sex	√	√	√	√	△	△
	Ethnicity	-	-	x	x	△	△
	Family history of CVD	√	√	√	√	-	-
	SBP	√	√	√	√	△	△
	Smoking	√	√	√	√	△	△
	Use of antihypertensive drugs	-	-	-	-	△	△
	TC	√	√	√	√	△	△
	HDL-C	√	√	√	√	△	△
	DM	√	√	√	√	△	△
	TGs	x	x	x	x	-	-
	Glucose	-	-	x	x	-	-
	CD4 count	x	x	√	√	△	△
	HIV RNA (HIV viral load)	x	x	x	x	△	△
	BMI	x	x	x	x	-	-
	Antiretroviral therapy	-	-	-	-	△	△
	Cumulative cART	-	-	x	x	-	-
	Cumulative PI exposure	x	x	√	x	△	△
	Cumulative NRTI exposure	-	-	√	x	-	-
	Exposure to lopinavir/ritonavir	√	√	x	x	-	-
	Exposure to indinavir	√	√	x	x	-	-
	Current abacavir use	√	√	√	x	-	-
	Lipodystrophy	x	x	x	x	-	-
	HIV exposure category	x	x	x	x	-	-
CVD endpoints	Carotid artery endarterectomy	Y	Y	Y	Y	N	N
	CHD death	Y	Y	Y	Y	N	N
	Invasive coronary artery procedure	Y	Y	Y	Y	N	N
	MI	Y	Y	Y	Y	Y	Y
	Stroke	Y	Y	Y	Y	N	N
Prediction horizon		5 years	10 years	5 years	5 years	10 years	10 years
Modeling methods		Poisson regression model	Poisson regression model	Cox model	Cox model	Lasso and ridge regression	Cox model

**(**√), predictor included in the final prediction model; (x), predictor not included in the final prediction model and excluded during modeling; (-), predictor was not considered before modeling; (△), predictor included in primary factor but did not report eventually included factor.

D:A:D, Data collection on Adverse Effects of Anti-HIV Drugs Study risk equation; CVD, cardiovascular disease; SBP, systolic blood pressure; TC, total cholesterol; HDL-C, high-density liptein cholesterol; DM, diabetes mellitus; TGs, triglycerides; HIV, human immunodeficiency virus; BMI, body mass index; cART, combination antiretroviral treatment; PI, protease inhibitor; NRTI, nucleoside reverse transcriptase inhibitor; CHD, coronary heart disease.

In total, 24 predictors were considered. Age, sex, family history of CVD, systolic blood pressure, Smoking, total cholesterol, high-density liptein cholesterol, and diabetes mellitus (*n* = 4) (33, 34, 39, 40) were the most common predictors, followed by current abacavir use (*n* = 3) (34, 39, 40). HIV-specific risk factors were included in four models: three models included current abacavir usage (33, 39, 40), two models included CD4 count (34) and exposure to lopinavir/ritonavir and indinavir (33, 39, 40), and one model included cumulative protease inhibitor (PI) exposure (34) and cumulative nucleoside reverse transcriptase inhibitor (NRTI) exposure (34).

The definitions of CVD outcomes revealed considerable heterogeneity. All six models included myocardial infarction (MI) (32–34, 39, 40), followed by coronary heart disease (CHD) death, carotid artery endarterectomy, invasive coronary artery procedure, and stroke (*n* = 4) (33, 34, 39, 40).

### 3.5. Meta-analysis of prediction models

**Figure 3:**
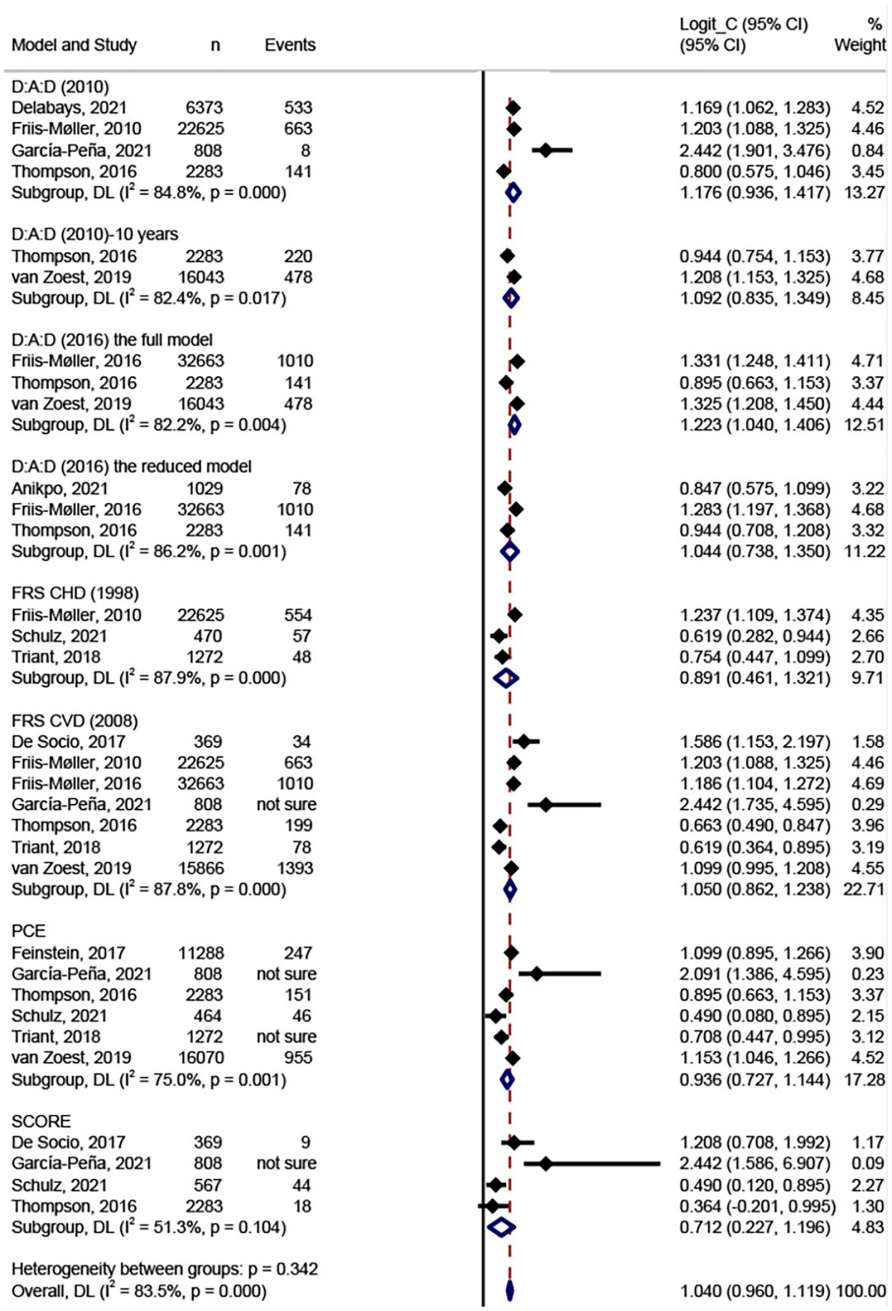
Meta-analysis of the C-statistic for the CVD prediction models in PLWH.

Figure 3 shows the results of the meta-analysis of the eight models. We conducted meta-analysis only on model discrimination. Calibrations were not synthesized due to inconsistent and inadequate data. In total, eight models were validated more than once and included in the meta-analysis. Two validation studies were removed from the meta-analysis because they did not report C-statistics or AUCs (36, 37). The FRS CVD model (2008) was the most widely validated model (*n* = 7) (20, 31, 33–35, 39, 40), followed by the PCE model (*n* = 6) (20, 32, 35, 38–40), the D:A:D model (2010) (*n* = 4) (19, 33, 35, 39), and the SCORE (*n* = 4) (31, 35, 38, 39). In the meta-analysis, the pooled estimated C-statistic/AUC was 0.76 (95% CI: 0.72–0.81, *I*^2^ = 84.8%) for the D:A:D (2010), 0.75 (95% CI: 0.70–0.79, *I*^2^ = 82.4%) for the D:A:D (2010) 10-year risk version, 0.77 (95% CI: 0.74–0.80, *I*^2^ = 82.2%) for the full D:A:D (2016) model, 0.74 (95% CI: 0.68–0.79, *I*^2^ = 86.2%) for the reduced D:A:D (2016) model, 0.71 (95% CI: 0.61–0.79, *I*^2^ = 87.9%) for the FRS CHD model (1998), 0.74 (95% CI: 0.70–0.78, *I*^2^ = 87.8%) for the FRS CVD model (2008), 0.72 (95% CI: 0.67–0.76, *I*^2^ = 75.0%) for the PCE model, and 0.67 (95% CI: 0.56–0.77, *I*^2^ = 51.3%) for the SCORE. However, there were no significant differences between any of the model pairings. Table 3 shows the results of the meta-analysis of C-statistics and AUC respectively. Overall, all of the models showed an acceptable discrimination.

**Table 3:** Meta-analysis of the Harrel's C-statistic and AUC respectively of the CVD models for PLWH.

Models	Number of Studies using Harrel's-C statistic	Logit C-statistic	*I*^2^, %	Number of Studies using AUC	Logit_AUC	*I*^2^, %	Effect model
D:A:D (2010) (19, 33, 35, 39)	1	0.800 (0.564, 1.036)	0	3	1.277 (1.048, 1.506)	79.7	Random
D:A:D (2010)-10 years (39, 40)	2	1.092 (0.835, 1.349)	82.4				Random
D:A:D (2016) the full model (34, 39, 40)	3	1.223 (1.040, 1.406)	82.2				Random
D:A:D (2016) the reduced model (30, 34, 39)	3	1.044 (0.738, 1.350)	86.2				Random
FRS CHD (1998) (20, 33, 38)	1	0.754 (0.428, 1.080)	0	2	0.947 (0.343, 1.374)	91.3	Random
FRS CVD (2008) (20, 30, 33–35, 39, 40)	4	0.914 (0.670, 1.158)	92.2	3	1.440 (0.983, 1.896)	57.9	Random
PCE (20, 32, 35, 38–40)	4	0.993 (0.809, 1.177)	72.6	2	1.095 (−0.427, 2.616)	72.2	Random
SCORE (31, 35, 38, 39)	1	0.364 (−0.201, 0.962)	0	3	0.914 (0.195, 1.633)	61.4	Random
Total	19	1.006 (0.912, 1.101)	86.6	13	1.124 (0.963, 1.285)	77.1	Random

AUC, area under the curve; D:A:D, Data collection on Adverse Effects of Anti-HIV Drugs Study risk equation; FRS, Framingham Risk Score; SCORE, Systematic COronary Risk Evaluation; PCE, pooled cohort equations of the American Heart Society/American score.

### 3.6. Subgroup analysis

To explore potential inequities in prediction models and sources of heterogeneity, we conducted subgroup analyses for sample size, age, and follow-up period (Table 4). The discrimination of PCE was significantly better in the group aged ≤40 years than in the groups aged 40–45 years (*P *= 0.024) and ≥45 years (*P *= 0.010). The discrimination of the full D:A:D (2016) model was significantly better in the group with sample size ≥10,000 than in the group with sample size 1,000–10,000 (*P *= 0.0076).

**Table 4:** Subgroup analysis of the C-statistics of the CVD prediction models for PLWH.

Subgroup		Number of Studies	Logit C-statistic (95% CI)	Model	*I*^2^, %	*P* for heterogeneity	*P* for trend
**Sample Size**							
D:A:D (2010)	≤1,000	1	2.442 (1.654, 3.230)	Random			0.2104
	1,000–10,000	2	1.000 (0.639, 1.360)	Random	87.1	0.005	
	≥10,000	1	1.203 (1.084, 1.321)	Random			
D:A:D (2016) the full model	1,000–10,000	1	0.895 (0.650, 1.140)	Random			0.0076*
	≥10,000	2	1.329 (1.262, 1.397)	Random	0	0.936	
D:A:D (2016) the reduced model	1,000–10,000	2	0.898 (0.717, 1.079)	Random	0	0.6	0.1359
	≥10,000	1	1.283 (1.197, 1.368)	Random			
FRS CVD (2008)	≤1000	2	1.744 (1.093, 2.396)	Random	17.7	0.27	0.0263*
	1,000–10,000	2	0.649 (0.501, 0.797)	Random	0	0.788	
	≥10,000	3	1.164 (1.105, 1.224)	Random	5.2	0.348	
PCE	≤1,000	2	1.095 (−0.427, 2.616)	Random	72.2	0.058	0.7699
	1,000–10,000	2	0.812 (0.629, 0.995)	Random	0	0.319	
	≥10,000	2	1.139 (1.044, 1.234)	Random	0	0.624	
SCORE	≤1,000	3	0.914 (0.195, 1.633)	Random	61.4	0.075	0.4669
	1,000–10,000	1	0.364 (−0.234, 0.962)	Random			
**Age**							
D:A:D (2010)	≤40	2	1.759 (0.551, 2.967)	Random	89.2	0.002	0.324
	40–45	2	1.000 (0.639, 1.360)	Random	87.1	0.005	
D:A:D (2016) the full model	≤40	1	1.331 (1.250, 1.412)	Random			0.6590
	40–45	2	1.124 (0.703, 1.544)	Random	89.5	0.002	
FRS CVD (2008)	≤40	3	1.199 (1.099, 1.300)	Random	32.9	0.225	0.4033
	40–45	3	1.051 (0.659, 1.444)	Random	90.8	<0.0001	
	≥45	1	0.619 (0.354, 0.885)	Random			
PCE	≤40	1	2.091 (0.487, 3.695)	Random			0.0078*
	40–45	2	1.083 (0.951, 1.215)	Random	43.7	0.169	
	≥45	1	0.640 (0.413, 0.868)	Random	0	0.384	
SCORE	40–45	2	0.778 (−0.049, 1.605)	Random	71.9	0.059	0.3616
	≥45	1	0.490 (0.103, 0.877)	Random			
**Follow-up**							
FRS CVD (2008)	≤5 years	2	0.924 (0.352, 1.495)	Random	93.5	<0.0001	0.3445
	5–10 years	3	0.996 (0.750, 1.242)	Random	92.6	<0.0001	
PCE	≤5 years	3	0.797 (0.436, 1.159)	Random	80.3	0.006	0.3763
	5–10 years	2	1.048 (0.800, 1.297)	Random	71.8	0.06	
**Models validated in North America**				44.9	0.046	0.1852	
D:A:D (2010)		1	0.690 (0.640, 0.740)	Random			
D:A:D (2010) 10-year version		1	0.720 (0.680, 0.760)	Random			
D:A:D (2016) the full model		1	0.710 (0.660, 0.760)	Random			
D:A:D (2016) the reduced model		2	0.711 (0.674, 0.748)	Random	0	0.598	
FRS CHD (1998)		1	0.680 (0.610, 0.750)	Random			
FRS CVD (2008)		2	0.657 (0.624, 0.690)	Random	0	0.786	
PCEs		3	0.715 (0.669, 0.761)	Random	63.8	0.063	
SCORE		1	0.590 (0.450, 0.730)	Random			
**Models in mainly white populations**				83.1	<0.001	0.2990	
D:A:D (2010)		2	0.733 (0.656, 0.810)	Random	87.7	0.004	
DAD (2010) 10-year version		2	0.749 (0.700, 0.797)	Random	81	0.022	
D:A:D (2016) the full model		3	0.775 (0.745, 0.797)	Random	79	0.009	
D:A:D (2016) the reduced model		2	0.756 (0.695, 0.817)	Random	82.2	0.018	
FRS CHD (1998)		2	0.733 (0.641, 0.826)	Random	84.3	0.012	
FRS CVD (2008)		5	0.728 (0.694, 0.762)	Random	89.3	<0.001	
PCE		4	0.731 (0.696, 0.765)	Random	70.2	0.018	
SCORE		1	0.590 (0.450, 0.730)	Random			

D:A:D, Data collection on Adverse Effects of Anti-HIV Drugs Study risk equation; FRS, Framingham Risk Score; SCORE, Systematic COronary Risk Evaluation; PCE, pooled cohort equations of the American Heart Society/American score.

* *P* values <0.05.

We conducted subgroup analysis to compare different models in North America (20, 30, 39) and Mainly White (20, 33, 34, 39, 40) population separately because all models were validated in these two groups. No significant differences were found (*P* > 0.05). In both groups, only the SCORE performed poorer than the other models in terms of discrimination, with a C-statistic of 0.59 (0.45, 0.73). No models were developed or validated in Sub-Saharan Africa and the Asia region.

## 4. Discussion

This systematic review identified 17 prediction models for predicting CVD risk among PLWH. Among them, six were HIV-specific models. All included studies were rated as having a high risk of bias against the PROBAST checklist. Only eight models were externally validated at least once. These were included in the meta-analysis, and all of them showed acceptable discrimination. The FRS was the most widely validated model. Most prediction models had acceptable discrimination. The full D:A:D (2016) model performed the best in terms of discrimination, followed by D:A:D (2010) and PCE. However, there were no significant differences between any of the model pairings. We also found that differences in model discrimination existed when they were stratified by geographic region, sample size, and age.

The full D:A:D (2016) model had higher discrimination for PLWH. However, it remained at a high risk of bias as they only included participants with complete information on all predictors. It is inappropriate to exclude participants with missing data directly. Missing data could be divided into missing completely at random (MCAR), missing at random (MAR), and missing not at random (MNAR) (41). MCAR and MAR are less problematic, but they seldomly occur (24, 41). Consequently, complete case analysis could lead to selection bias, and the characteristics of participants may differ from those with missing data. A nonrandom sample subset could frequently generate considerable bias that cannot be overcome in estimating model parameters and yielding predictive performance (24, 41, 42). Currently, common strategies for handling missing data include zero imputation, mean imputation, and multiple imputation (43). Multiple imputation is often regarded as a preferred method for avoiding selection bias and statistical power loss (24, 44). Furthermore, it is advisable to consider combining unsupervised and supervised learning methods for imputation (42). Strategies for handling missing data should be chosen with caution depending on the intended application of the prediction model (41).

The D:A:D cohort was the largest HIV cohort in our study. A large sample size was one of the key points in developing robust models that would be more reliable for application in new datasets (45). In contrast, nearly half of the included studies (*n* = 7) (20, 30, 31, 35–38) had limited sample sizes and numbers of outcome events. For development studies, the sample size should ensure at least 10 events per candidate predictor parameter. For studies validating prediction models, a minimum of 100 events and 100 nonevents are suggested (24). A small sample size may result in model overfitting and inaccurate predictions (45, 46).

Current models applied for PLWH lack HIV-specific predictors. This might be attributed to the fact that the majority of prediction models are developed for the general population. According to our review, frequently used traditional CVD risk predictors included age, smoking status, systolic blood pressure, total cholesterol, and diabetes mellitus. Common HIV-specific predictors included abacavir usage, CD4 count, and exposure to lopinavir/ritonavir and indinavir. Previous studies presented multiple factors that drive the risk of CVD among PLWH (47). Decreased CD4 T-cell counts and increased HIV RNA have been demonstrated to be linked to increased CVD rates (47, 48). A persistently lower or inverted CD4/CD8 ratio has emerged as an independent predictor of CVD risk (48). Analysis from observational studies showed that most PIs, such as ritonavir-boosted darunavir/ritonavir, are associated with progressively increased CVD risk, and the effect is cumulative (49, 50). The results of D:A:D cohort indicated that the incidence rate ratio of using ritonavir-boosted darunavir was 1.59 (95% CI: 1.33–1.91, per 5 years of additional use) compared to non-use (50). Our review indicated that the full D:A:D model (2016) performed better than the reduced model (C-statistics: 0.775 vs. 0.739). This might be due to the reduced model leaving out ART covariates, such as PI and NRTI exposure. We recommend including CD4 counts and the CD4/CD8 ratio, as well as ART covariates (PI and NRTI exposure) as potential predictors in future studies that aim to develop HIV-specific models. In addition, it is critical to understand the role of HIV-specific inflammation and immune activation in conferring CVD risk (48, 51–53). Few models contained immunological-related factors, such as soluble markers of interleukin-1 (IL-1) (53). The feasibility and effectiveness of adding immunological-related factors into models still remain to be assessed. Further study is needed to add HIV-specific predictors to models.

Among published models, most used univariate or multivariate regression analysis and no validation process. The performance of the prediction model would be overestimated if there was no internal or external validation process (54). Internal validation is beneficial to provide a more accurate assessment of model performance in new subjects (54). External validation is necessary to determine a model's reproducibility and generalizability to populations with different characteristics (55). Models that have not been validated should not be recommended for clinical use. We suggest that future studies validate the multiple existing HIV-specific models and compare their performance head to head.

Calibration, one of the important aspects of validating model performance, has remained underreported in many published studies. Calibration refers to the accuracy of absolute risk estimates by comparing how similar the predicted risk to the true (observed) risk in different risk strata (56). It could be assessed in various ways, including the Hosmer–Lemeshow test (H–L test), calibration-in-the-large, Brier score, Cox intercept and slope, and integrated calibration index (57, 58). Although the H–L test was one of the most common proxies for calibration measures, we should consider its limitations, including its vulnerability when increasing the sample size and the arbitrary number of groups (55, 57). We recommend that future studies combine multiple calibration measures to assess the model calibration more comprehensively and to promote comparability of model calibration across studies.

Our subgroup analysis revealed differences in the model's discrimination when stratified by age. We found that the model's discrimination degraded with age. Age is an important independent risk factor for CVD (59). Aging is always accompanied by changes in the heart and vascular system. In addition, older individuals are more susceptible to drug interactions and side effects (60). Frailty, a common symptom in older people, may also be associated with an increased risk of CVD and CVD mortality (61, 62). These characteristics of older adults may lead to changes in the relationship between risk factors and CVD outcomes. Specific CVD risk models for older PLWH should be developed and validated in the future. In addition, the majority of models were developed and validated in America and Europe, whereas there was a lack of models from Asia and Africa. As global statistics revealed, Sub-Saharan Africa and the Asia have the highest burden of HIV-associated CVD (63). Models should be adapted to these regions and validated further in the future due to differences in race/ethnicity, healthcare systems, and lifestyle.

## 5. Limitations

There are several limitations of our study. First, we included only studies that were in English or Chinese. Articles in other languages should be summarized in future reviews. Second, most data for model development and validation came from developed countries. Caution should be used when applying our findings to individuals from different regions. Third, we conducted a meta-analysis only on discrimination rather than calibration. This is because of the inadequate reporting of calibration in the validation studies.

## 6. Implications for practice

The full D:A:D (2016) model had higher discrimination for PLWH. In addition, the findings indicated that model performance was associated with age. In clinical practice, health professionals should revalidate models if the population, region, or age to which they are applied is different from the original study. Moreover, only eight models were validated more than once. Models that have not been thoroughly validated are not useful for clinical practice. Health professionals should focus on developing, updating, and validating HIV-specific models and report them in accordance with the Transparent Reporting of a multivariable prediction model for Individual Prognosis Or Diagnosis (TRIPOD) guidelines. Specific CVD risk models for older PLWH, as well as models for Sub-Saharan Africa and the Asia region should be established that CVD risk and prevalence vary in different regions due to culture, local economy and health care policy.

## 7. Conclusion

Our systematic review summarized the prediction models for CVD in PLWH and conducted a meta-analysis on model discrimination performance. All of the models showed acceptable discrimination. The full D:A:D (2016) model performed the best in terms of discrimination, followed by D:A:D (2010) and PCE. However, all these models were assessed as having a high risk of bias. Future studies should adhere to the TRIPOD guidelines to ensure the quality and applicability of the models. Researchers should focus on developing and validating CVD models for PLWH. Specific CVD risk models for older PLWH, as well as models for Sub-Saharan Africa and the Asia region should be established.

## Data Availability

The original contributions presented in the study are included in the article/**Supplementary Material**, further inquiries can be directed to the corresponding author/s.
