# Supplementary material for: Prediction models for cardiovascular disease risk among people living with HIV: A systematic review and meta-analysis
Source: Front Cardiovasc Med. 2023 Mar 23;10:1138234. doi: 10.3389/fcvm.2023.1138234 (PMC10077152; doi:10.3389/fcvm.2023.1138234)
Supplement: Supplementary file 1 [file Table1.docx]

**Supplementary Material**

**Prediction Models for Cardiovascular Disease Risk Among People Living with HIV: A Systematic Review and Meta-analysis**

**Appendix file 1 Searching strategies and results**

**PubMed / MEDLINE**

**Search time: 2022-01-14**

| **Search** | **Query** | **Items found** |
| --- | --- | --- |
| #1 | HIV [MeSH] OR HIV-1 [MeSH] OR HIV-2 [MeSH] OR acquired immunodeficiency syndrome [MeSH] OR HIV infections [MeSH] | 334786 |
| #2 | "HIV"[Title/Abstract] OR "HIV infect*"[Title/Abstract] OR “HIV-positive” [Title/Abstract] OR "AIDS"[Title/Abstract] OR "HIV/AIDS"[Title/Abstract] OR "human immunodeficiency virus*"[Title/Abstract] OR "human immunedeficiency virus*"[Title/Abstract] OR "human immune-deficiency virus*"[Title/Abstract] OR "acquired immunodeficiency syndrome"[Title/Abstract] OR "acquired immunedeficiency syndrome"[Title/Abstract] OR "acquired immune-deficiency syndrome"[Title/Abstract] OR "PLWHA"[Title/Abstract] OR "PLWH"[Title/Abstract] OR "PLHIV"[Title/Abstract] OR "people living with HIV"[Title/Abstract] | 436331 |
| #3 | #1 OR #2 | 473363 |
| #4 | prognoses [MeSH] OR risk [MeSH] OR risk assessment [MeSH] OR risk factors [MeSH] OR heart disease risk factors [MeSH] OR cardiometabolic risk factors [MeSH] OR epidemiological models [MeSH] | 2848442 |
| #5 | progn*[Title/Abstract] OR risk[Title/Abstract] OR predict*[Title/Abstract] OR model*[Title/Abstract] OR score*[Title/Abstract] OR factor*[Title/Abstract] OR “risk factor*” [Title/Abstract] OR "predict* model*"[Title/Abstract] OR "prognostic model*"[Title/Abstract] OR "risk prediction"[Title/Abstract] OR "risk score*"[Title/Abstract] OR "risk index"[Title/Abstract] OR “risk assessment*” [Title/Abstract] OR "risk calculat*"[Title/Abstract] OR “risk appraisal*” [Title/Abstract] OR “prediction equation*” [Title/Abstract] OR “risk equation*” [Title/Abstract] OR clinical*[Title/Abstract] OR "epidemiological model*"[Title/Abstract] OR "parametric model*"[Title/Abstract] OR framework*[Title/Abstract] OR "prediction indices"[Title/Abstract] OR "prediction rule*"[Title/Abstract] OR rule*[Title/Abstract] | 11833832 |
| #6 | #4 OR #5 | 12659344 |
| #7 | "machine learning"[Title/Abstract] OR "artificial intelligence"[Title/Abstract] OR "algorithm"[Title/Abstract] OR "score"[Title/Abstract] OR "deep learning"[Title/Abstract] OR "AUC"[Title/Abstract] OR "area under the curve"[Title/Abstract] OR "area under the receiver operator characteristic curve"[Title/Abstract] OR "ROC"[Title/Abstract] OR "receiver operating characteristic"[Title/Abstract] OR discrimination[Title/Abstract] OR calibration[Title/Abstract] OR sensitivity[Title/Abstract] OR specificity[Title/Abstract] OR multivariable[Title/Abstract] OR indices[Title/Abstract] OR stratification[Title/Abstract] OR "logistic regression"[Title/Abstract] OR "poisson regression model"[Title/Abstract] OR "cox regression model"[Title/Abstract] OR "cox regression"[Title/Abstract] OR cox[Title/Abstract] OR "cox’s proportional hazard regression"[Title/Abstract] OR "cox proportional hazards model"[Title/Abstract] OR "Kaplan-Meier curves"[Title/Abstract] OR "Kaplan-Meier plot"[Title/Abstract] OR Kaplan-Meier[Title/Abstract] | 2963555 |
| #8 | “cardiovascular disease”[Title/Abstract] OR CVD[Title/Abstract] OR “cardiovascular event*”[Title/Abstract] OR cardiovascular[Title/Abstract] OR CV[Title/Abstract] OR “coronary heart disease”[Title/Abstract] OR CHD[Title/Abstract] OR “heart disease”[Title/Abstract] OR “myocardial infarction*”[Title/Abstract] OR MI[Title/Abstract] OR stroke[Title/Abstract] OR “invasive coronary artery procedure*”[Title/Abstract] | 1094293 |
| #9 | #3 AND #6 AND #7 AND #8 | 1816 |
| #10 | #3 AND #6 AND #7 AND #8 Filters: English, from 1996 - 2022 | 1757 |

**CINAHL (EBSCO)**

**Search time: 2022-01-14**

| **Search** | **Query** | **Items found** |
| --- | --- | --- |
| S1 | (MH "Human Immunodeficiency Virus+") OR (MH "HIV-Positive Persons+") OR (MH "HIV Infections+") | 104027 |
| S2 | TI “HIV” OR “HIV infect*” OR “HIV-positive” OR “AIDS” OR “HIV/AIDS” OR “human immunodeficiency virus*” OR “human immunedeficiency virus*” OR “human immune-deficiency virus*” OR “acquired immunodeficiency syndrome” OR “acquired immunedeficiency syndrome” OR “acquired immune-deficiency syndrome” OR “PLWHA” OR “PLWH” OR “PLHIV” OR “people living with HIV” | 88368 |
| S3 | S1 OR S2 | 121717 |
| S4 | (MH "Prognosis+") OR (MH “risk factors”) OR (MH “cardiovascular risk factors”) | 686100 |
| S5 | TI progn* OR risk OR predict* OR model* OR score* OR factor* OR “risk factor*” OR “predict* model*” OR “prognostic model*” OR “risk prediction” OR “risk score*” OR “risk index” OR “risk assessment” OR “risk calculat*” OR “risk appraisal” OR “risk equation*” OR clinical OR “epidemiological model*” OR “parametric model*” OR framework* OR “prediction indices” OR “prediction rule*”  OR rule* | 874155 |
| S6 | S4 OR S5 | 1384846 |
| S7 | TI "machine learning" OR "artificial intelligence" OR "algorithm" OR "score" OR "deep learning" OR "AUC" OR "area under the curve" OR "area under the receiver operator characteristic curve" OR "ROC" OR "receiver operating characteristic" OR discrimination OR calibration OR sensitivity OR specificity OR multivariable OR indices OR stratification OR “logistic regression” OR “poisson regression model” OR “cox regression model” OR “cox regression” OR cox OR “cox’s proportional hazard regression” OR “cox proportional hazards model” OR “Kaplan-Meier curves” OR “Kaplan-Meier plot” OR Kaplan-Meier | 94256 |
| S8 | AB "machine learning" OR "artificial intelligence" OR "algorithm" OR "score" OR "deep learning" OR "AUC" OR "area under the curve" OR "area under the receiver operator characteristic curve" OR "ROC" OR "receiver operating characteristic" OR discrimination OR calibration OR sensitivity OR specificity OR multivariable OR indices OR stratification OR “logistic regression” OR “poisson regression model” OR “cox regression model” OR “cox regression” OR cox OR “cox’s proportional hazard regression” OR “cox proportional hazards model” OR “Kaplan-Meier curves” OR “Kaplan-Meier plot” OR Kaplan-Meier | 734138 |
| S9 | S7 OR S8 | 773254 |
| S10 | S6 OR S9 | 1859569 |
| S11 | (MH " Cardiovascular diseases ") | 56631 |
| S12 | TI “cardiovascular disease” OR CVD OR “cardiovascular event*” OR cardiovascular OR CV OR “coronary heart disease” OR CHD OR “heart disease” OR “myocardial infarction*” OR MI OR “coronary heart disease” OR stroke OR “invasive coronary artery procedure*” | 155402 |
| S13 | AB “cardiovascular disease” OR CVD OR “cardiovascular event*” OR cardiovascular OR CV OR “coronary heart disease” OR CHD OR “heart disease” OR “myocardial infarction*” OR MI OR “coronary heart disease” OR stroke OR “invasive coronary artery procedure*” | 236043 |
| S14 | S11 OR S12 OR S13 | 324216 |
| S15 | S3 AND S10 AND S14 | 1180 |
| S16 | S15 English, 19960101-20221231 | 1168 |

**EMBASA(OVID)**

**Search time: 2022-01-14**

| **Search** | **Query** | **Items found** |
| --- | --- | --- |
| #1 | exp Human immunodeficiency virus/ | 207754 |
| #2 | (HIV or HIV infect* or human immunodeficiency virus* or HIV-positive or human immunedeficiency virus* or human immune-deficiency virus* or acquired immunodeficiency syndrome or acquired immunedeficiency syndrome or acquired immune-deficiency syndrome or PLWHA or PLWH or PLHIV or people living with HIV).m_titl. | 302740 |
| #3 | #1 or #2 | 381307 |
| #4 | exp prognosis/ or exp risk factor/ or exp cardiovascular risk factor/ or exp cardiovascular risk/ | 2101968 |
| #5 | (progn* or risk or predict* or model* or score* or factor* or risk factor* or predict* model* or prognostic model* or risk prediction or risk score* or risk assessment or risk appraisal or risk equation or prediction indices or prediction rule*).m_titl. | 2888844 |
| #6 | (machine learning or algorithm or AUC or area under the curve or area under the receiver operator characteristic curve or ROC or receiver operating characteristic or discrimination or calibration or sensitivity or specificity or indices or logistic regression or poisson regression model or cox regression or cox or cox proportional hazards model or Kaplan-Meier curves or Kaplan-Meier).m_titl. | 299019 |
| #7 | #5 and #6 | 39048 |
| #8 | #4 or #7 | 2133978 |
| #9 | (cardiovascular disease or CVD or cardiovascular event* or cardiovascular or CV or coronary heart disease or CHD or heart disease or myocardial infarction* or MI or coronary heart disease or stroke or invasive coronary artery procedure*).m_titl. | 552145 |
| #10 | #3 and #8 and #9 | 1600 |
| #11 | limit 10 to (english language and yr="1996 -Current") | 1517 |

**MEDLINE(OVID)**

**Search time: 2022-01-14**

| **Search** | **Query** | **Items found** |
| --- | --- | --- |
| #1 | exp Human immunodeficiency virus/ | 104041 |
| #2 | (HIV or HIV infect* or human immunodeficiency virus* or HIV-positive or human immunedeficiency virus* or human immune-deficiency virus* or acquired immunodeficiency syndrome or acquired immunedeficiency syndrome or acquired immune-deficiency syndrome or PLWHA or PLWH or PLHIV or people living with HIV).m_titl. | 227371 |
| #3 | #1 or #2 | 246644 |
| #4 | exp prognosis/ or exp Risk Factors/ or exp Cardiometabolic Risk Factors/ or exp Heart Disease Risk Factors/ or exp Risk Assessment/ or exp Risk/ or exp Models, Cardiovascular/ | 2872914 |
| #5 | (progn* or risk or predict* or model* or score* or factor* or risk factor* or predict* model* or prognostic model* or risk prediction or risk score* or risk index or risk calculat* or risk assessment or risk appraisal or risk equation or clinical or epidemiological model* or parametric model* or framework* or prediction indices or prediction rule* or rule*).m_titl. | 2726078 |
| #6 | (machine learning or artificial intelligence or algorithm or score or deep learning or AUC or area under the curve or area under the receiver operator characteristic curve or ROC or receiver operating characteristic or discrimination or calibration or sensitivity or specificity or multivariable or indices or stratification or logistic regression or poisson regression model or cox regression model or cox regression or cox or cox proportional hazards model or Kaplan-Meier curves or Kaplan-Meier plot or Kaplan-Meier).m_titl. | 282366 |
| #7 | #4 or #5 or #6 | 4963115 |
| #8 | (cardiovascular disease or CVD or cardiovascular event* or cardiovascular or CV or coronary heart disease or CHD or heart disease or myocardial infarction* or MI or coronary heart disease or stroke or invasive coronary artery procedure*).m_titl. | 359015 |
| #9 | #3 and #7 # and #8 | 864 |
| #10 | limit 9 to (english language and yr="1996 -Current") | 803 |

**Web of Science**

**Search time: 2022-01-14**

| **Search** | **Query** | **Items found** |
| --- | --- | --- |
| **#1** | **TI=(“HIV” OR “HIV infect*” OR HIV-positive OR “AIDS” OR “HIV/AIDS” OR “human immunodeficiency virus*” OR “human immunedeficiency virus*” OR “human immune-deficiency virus*” OR “acquired immunodeficiency syndrome” OR “acquired immunedeficiency syndrome” OR “acquired immune-deficiency syndrome” OR “PLWHA” OR “PLWH” OR “PLHIV” OR “people living with HIV”)** | **458986** |
| **#2** | **TI=(progn* OR predict* OR risk OR model* OR score* OR factor* OR risk factor* OR “predict* model*” OR “prognostic model*” OR “risk prediction” OR “risk score*” OR “risk index” OR “risk calculat*” OR “risk assessment” OR risk appraisal OR prediction equation* OR “epidemiological model*” OR “parametric model*” OR framework* OR “prediction indices” OR “prediction rule*”)** | **7478883** |
| **#3** | **TS=(“machine learning” OR “artificial intelligence” OR “algorithm” OR “score” OR “deep learning” OR “AUC” OR “area under the curve” OR “area under the receiver operator characteristic curve” OR “ROC” OR “receiver operating characteristic” OR discrimination OR calibration OR sensitivity OR specificity OR multivariable OR indices OR stratification OR "logistic regression" OR "poisson regression model" OR "cox regression model" OR "cox regression" OR cox OR "cox’s proportional hazard regression" OR "cox proportional hazards model" OR "Kaplan-Meier curves" OR "Kaplan-Meier plot" OR Kaplan-Meier)** | **12371758** |
| **#4** | **TS=(“cardiovascular disease” OR CVD OR “cardiovascular event*” OR cardiovascular OR CV OR “coronary heart disease” OR CHD OR “heart disease” OR “myocardial infarction*” OR MI OR “coronary heart disease” OR stroke OR “invasive coronary artery procedure*”)** | **5219472** |
| **#5** | **#1 AND #2 AND #3 AND #4** | **1003** |
| **#6** | **#5: 1996-01-01 to 2022-01-14, English** | **947** |

**Appendix file 2 The results of risk of bias and applicability of the included studies**

|  | **ROB** | | | | | | | | | | | | | | | | | | | | **Applicability** | | |  | **Overall** | | | | | |
| --- | --- | --- | --- | --- | --- | --- | --- | --- | --- | --- | --- | --- | --- | --- | --- | --- | --- | --- | --- | --- | --- | --- | --- | --- | --- | --- | --- | --- | --- | --- |
|  | **Participants** | | **Predictors** | | | **Outcome** | | | | | | **Analysis** | | | | | | | | | **Participants** | **Predictors** | **Outcome** |  | **ROB** | | | | | **Applicability** |
| Signaling questions | 1.1 | 1.2 | 2.1 | 2.2 | 2.3 | 3.1 | 3.2 | 3.3 | 3.4 | 3.5 | 3.6 | 4.1 | 4.2 | 4.3 | 4.4 | 4.5 | 4.6 | 4.7 | 4.8 | 4.9 |  |  |  | Participants | | Predictors | Outcome | Analysis | All | All |
| Anikpo, 2021 | Y | N | PY | Y | Y | Y | Y | Y | Y | Y | Y | N | Y | Y | PN | NA | U | Y | N | Y | Y | Y | Y | ‐ | | + | + | - | ‐ | + |
| Delabays, 2021 | Y | Y | PY | Y | Y | Y | Y | Y | Y | Y | Y | Y | U | Y | Y | NA | U | PY | N | Y | Y | Y | Y | + | | + | + | - | ‐ | + |
| De Socio, 2017 | Y | Y | Y | Y | Y | Y | Y | Y | Y | Y | Y | N | U | Y | U | NA | U | Y | N | Y | Y | Y | Y | + | | + | + | - | ‐ | + |
| Feinstein, 2017 | Y | Y | PY | Y | Y | NY | NY | Y | NY | Y | Y | Y | U | Y | U | U | PN | Y | N | Y | Y | Y | Y | + | | + | + | - | ‐ | + |
| Friis-Moller, 2010 | Y | N | PN | Y | Y | Y | Y | Y | Y | Y | PY | Y | PY | Y | N | PY | U | Y | Y | Y | Y | Y | Y | ‐ | | ‐ | + | - | ‐ | + |
| Friis-Møller, 2016 | Y | N | PN | Y | Y | Y | Y | Y | Y | Y | Y | Y | Y | Y | PN | Y | U | Y | Y | Y | Y | Y | Y | ‐ | | ‐ | + | ? | - | + |
| García-Peña, 2021 | Y | N | U | Y | Y | PY | Y | Y | Y | Y | Y | N | U | N | N | NA | U | N | N | Y | Y | Y | Y | ‐ | | ? | + | - | ‐ | + |
| Herrera, 2016 | Y | Y | Y | Y | Y | Y | Y | Y | Y | Y | Y | N | U | Y | Y | NA | U | N | N | Y | Y | Y | Y | + | | + | + | - | ‐ | + |
| Raggi, 2016 | Y | Y | PY | Y | Y | Y | Y | Y | Y | Y | PY | N | U | Y | U | NA | U | N | N | Y | Y | Y | Y | + | | + | + | - | ‐ | + |
| Schulz, 2021 | Y | N | Y | Y | Y | Y | Y | Y | Y | Y | Y | N | U | N | N | NA | U | N | N | Y | Y | Y | Y | ‐ | | + | + | - | ‐ | + |
| Thompson-Paul, 2016 | Y | N | Y | Y | Y | Y | Y | Y | Y | Y | Y | Y | U | Y | N | NA | U | Y | U | Y | Y | Y | Y | ‐ | | + | + | - | ‐ | + |
| Triant, 2018 | Y | Y | Y | Y | Y | Y | Y | Y | Y | Y | Y | N | U | Y | Y | NA | U | Y | Y | Y | Y | Y | Y | + | | + | + | - | ‐ | + |
| van Zoest, 2019 | Y | PN | Y | Y | Y | Y | Y | Y | Y | Y | Y | Y | U | Y | PY | NA | U | Y | Y | Y | Y | Y | Y | ‐ | | + | + | - | ‐ | + |

**ROB = risk of bias, Y = yes, PY = probably yes, N = no, PN = probably no, U = no information, NA = not applicable, + indicate low risk of bias or low concern regarding applicability, - indicate high risk of bias or high concern regarding applicability, and ? indicate unclear risk of bias or unclear concern regarding applicability.**
